# Evolution and distribution of medullary bone: evidence from a new Early Cretaceous enantiornithine bird

**DOI:** 10.1093/nsr/nwz214

**Published:** 2019-12-19

**Authors:** Min Wang, Jingmai K O’Connor, Alida M Bailleul, Zhiheng Li

**Affiliations:** Key Laboratory of Vertebrate Evolution and Human Origins, Institute of Vertebrate Paleontology and Paleoanthropology, Chinese Academy of Sciences, Beijing 100044, China; CAS Center for Excellence in Life and Paleoenvironment, Beijing 100044, China; Key Laboratory of Vertebrate Evolution and Human Origins, Institute of Vertebrate Paleontology and Paleoanthropology, Chinese Academy of Sciences, Beijing 100044, China; CAS Center for Excellence in Life and Paleoenvironment, Beijing 100044, China; Key Laboratory of Vertebrate Evolution and Human Origins, Institute of Vertebrate Paleontology and Paleoanthropology, Chinese Academy of Sciences, Beijing 100044, China; CAS Center for Excellence in Life and Paleoenvironment, Beijing 100044, China; Key Laboratory of Vertebrate Evolution and Human Origins, Institute of Vertebrate Paleontology and Paleoanthropology, Chinese Academy of Sciences, Beijing 100044, China; CAS Center for Excellence in Life and Paleoenvironment, Beijing 100044, China

**Keywords:** Aves, Cretaceous, medullary bone, phylogeny, reproduction

## Abstract

Living birds are unique among vertebrates in the formation of a female-specific bone tissue called medullary bone (MB) that is strictly associated with reproductive activity. MB is a rapidly mobilized source of calcium and phosphorus for the production of eggshell. Among living taxa, its skeletal distribution can be highly extensive such that it even exists in the ribs of some species. Due to its ephemeral nature, MB is rarely fossilized and so little is understood with regard to the origin of MB and its skeletal distribution in early taxa. Here we describe a new Early Cretaceous enantiornithine bird, *Mirusavis parvus*, gen. et. sp. nov., indicating that skeleton-wide distribution of MB appeared early in avian evolution. We suggest that this represents the plesiomorphic condition for the Aves and that the distribution of MB observed among extant neornithines is a product of increased pneumatization in this lineage and natural selection for more efficient distribution of MB.

## INTRODUCTION

Birds are characterized by a unique suit of reproductive features, being one of the few extant vertebrates that exclusively reproduce through the production of external hard-shelled eggs [1,2] and having a single functional ovary [1]. One of the most unusual reproductive features of modern birds is the deposition of a female-specific endosteal bone tissue called medullary bone (MB) within the cavernous spaces of the skeleton in female birds during egg laying [3,4]. MB provides the majority of the calcium and phosphorus needed for eggshell formation [3,5,6] and can be used as a reliable indicator of reproductive activity in fossils preserving this tissue. Despite its highly ephemeral nature, MB has been reported in some non-avian dinosaurs and Early Cretaceous birds [6–11], suggesting that this ‘avian’ reproductive tissue evolved early in the Dinosauria and was widely distributed within the dinosaurian phylogeny. However, identification of MB in fossils is hindered by incomplete preservation and the existence of pathologies that superficially resemble MB [12]; currently, the best available data limit MB to Aves or the Theropoda [7]. Here we describe a new enantiornithine, *Mirusavis parvus*, gen. et. sp. nov., from the Lower Cretaceous period, based on a partial skeleton that preserves MB in nearly all preserved elements, including the cervical vertebrae, ribs and humerus—elements commonly devoid of MB in living birds. We suggest that this represents the plesiomorphic condition for the Aves and that differences in distribution observed among extant neornithines probably evolved from a complex interplay between developmental strategy, body size, ecology and skeletal pneumaticity.

## RESULTS

### Systematic paleontology

Aves Linnaeus, 1758 [13]
Ornithothoraces Chiappe, 1995 [14]
Enantiornithes Walker, 1981 [15]
*Mirusavis* gen. nov.
Diagnosis. As for species.
*Mirusavis parvus* sp. nov.

#### Holotype

**Figure 1.**
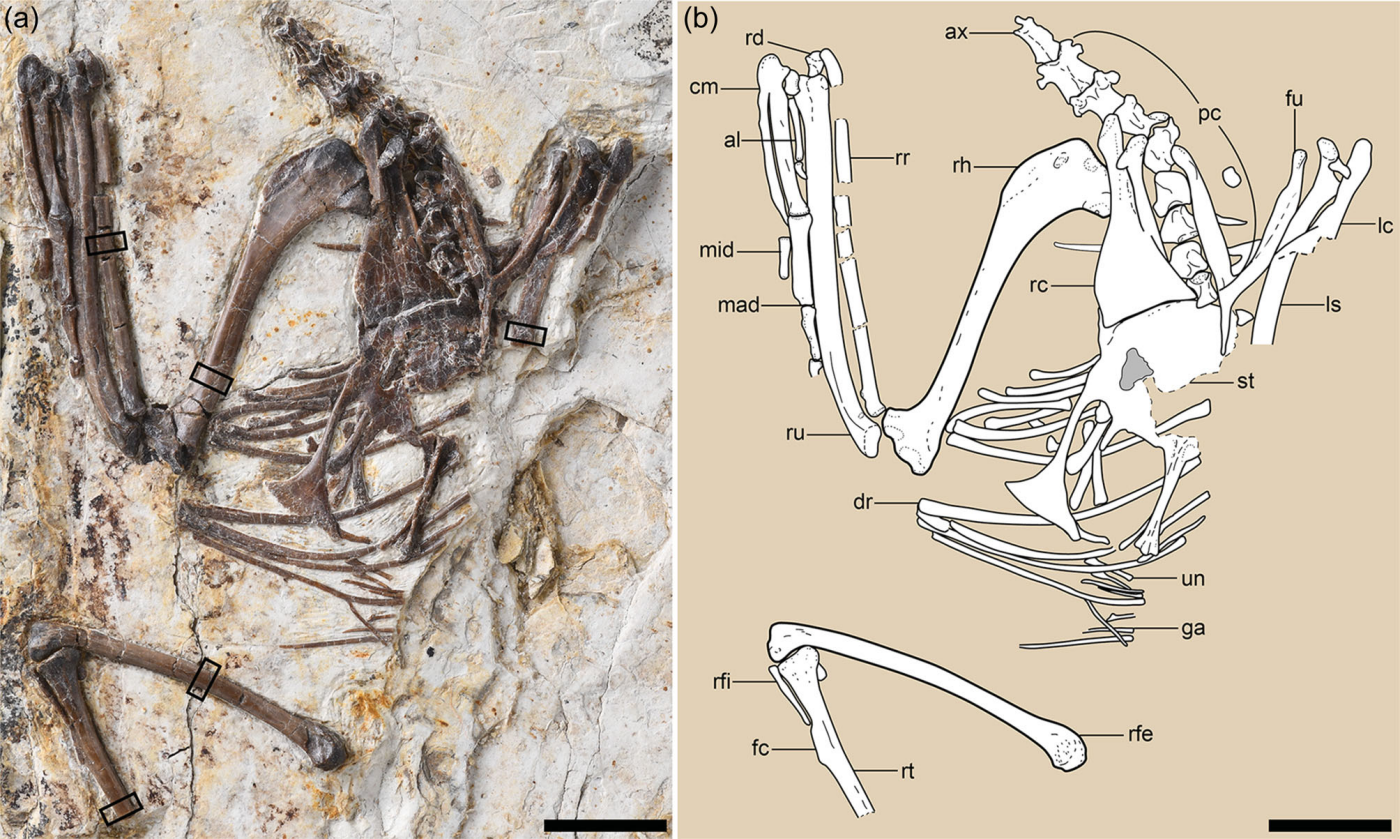
*Mirusavis parvus*, holotype, IVPP V18692. (a) Photograph and (b) line drawing. al, alular digit; ax, axis; cm, carpometacarpus; dr, dorsal rib; fc, fibular crest; fu, fucula; ga, gastralia; lc, left coracoid; ls, left scapula; mad, major digit; mid, minor digit; pc, post-axis cervical vertebrae; rc, right coracoid; rd, right radiale; rfe, right femur; rfi, right fibula; rh, right humerus; rr, right radius; ru, right ulna; st, sternum; un, uncinate process. The boxes denote the positions from which the samples were taken for histological analysis. Scale bars equal 10 mm.

IVPP (Institute of Vertebrate Paleontology and Paleoanthropology) V18692 is an articulated partial skeleton ventrally exposed in a single slab preserving most of the cervical vertebrae, the pectoral girdle, the complete right forelimb and a partial right hindlimb (Fig. 1; see Supplementary Table 1).

#### Etymology

‘*Mirus*’ (‘unexpected’ in Latin) and ‘*avis*’ (‘bird’ in Latin) refer to the serendipitous discovery of a Mesozoic bird preserved during the laying cycle; ‘*parvus*’ means ‘small’ in Latin.

#### Diagnosis

A small enantiornithine is distinguishable from other enantiornithines based on the unique combination of the following features: sternum with craniolateral processes; elongated intermediate trabecula of the sternum caudolaterally directed; reduced humeral deltopectoral crest; distal margin of humerus strongly angled caudoventrally; ulna with well-developed bicipital tubercle and olecranon process; dorsal cotyle of ulna convex; radius with bicipital tubercle; interosseous surface of radius smooth; and ungual of major digit larger than that of the alular digit (see the ‘Materials and methods’ section for differential diagnosis).

#### Locality and horizon

Near Sihedang Town, Lingyuan City, Liaoning Province; Lower Cretaceous Yixian Formation, Jehol Group [16].

### Anatomical description

Limb-bone cross sections show the presence of the inner circumferential layer but the absence of the outer circumferential layer [17], indicating that IVPP V18692 is a subadult female that died during egg laying (see the ‘Osteological description’ section bellow). Using an empirical equation based on the linear relationship between the femoral circumference and the body mass (see the ‘Materials and methods’ section) [18], IVPP V18692 is estimated to have had a body mass of approximately 16 g. The post-axial cervicals are keeled ventrally and lack carotid processes (Fig. 1a). The cranial and caudal articular facets of the caudal four cervicals are weakly concave, indicating the series was not heterocoelous. In contrast, partial or complete heterocoely is present in some other enantiornithines such as *Piscivorenantiornis* and *Pengornis* [19].

The Y-shaped furcula has an interclavicular angle of 50° (Supplementary Fig. 1a and b). The hypocleidium is mediolaterally compressed, approaching one-third of the length of the ramus, proportionally longer than in *Shangyang* and *Shanweiniao*, but shorter than that of *Pterygornis* (Supplementary Fig. 1c–e). The incomplete scapulae have flat medial surfaces. As in other enantiornithines [20], the strut-like coracoid has a straight acrocoracoid process and lacks a procoracoid process. The sternum remains in articulation with the right coracoid via the coracoidal sulcus (Supplementary Fig. 1a). The medial angle of the coracoid terminates short of the midpoint of the cranial margin of the sternum, indicating that the coracoidal sulci are separated mediolaterally. The craniolateral process extends craniolaterally from both the cranial and lateral margins of the sternum. This structure is absent in most enantiornithines except *Concornis* and *Shangyang* [19,21]. The lateral trabecula is elongate, projects caudally parallel to the longitudinal axis of the sternum and ends in a large, asymmetrical, triangular expansion, reminiscent of the condition in *Shangyang* and *Pterygornis* [21], although the shaft proximal to the distal expansion is markedly slender compared to the latter two taxa. The intermediate trabecula is straight, pointed and mostly caudally oriented with a slight lateral deflection, whereas, in other enantiornithines, this process is typically proximally broad, triangular and medially curved (Supplementary Fig. 1c–f) [19,21].

**Figure 2.**
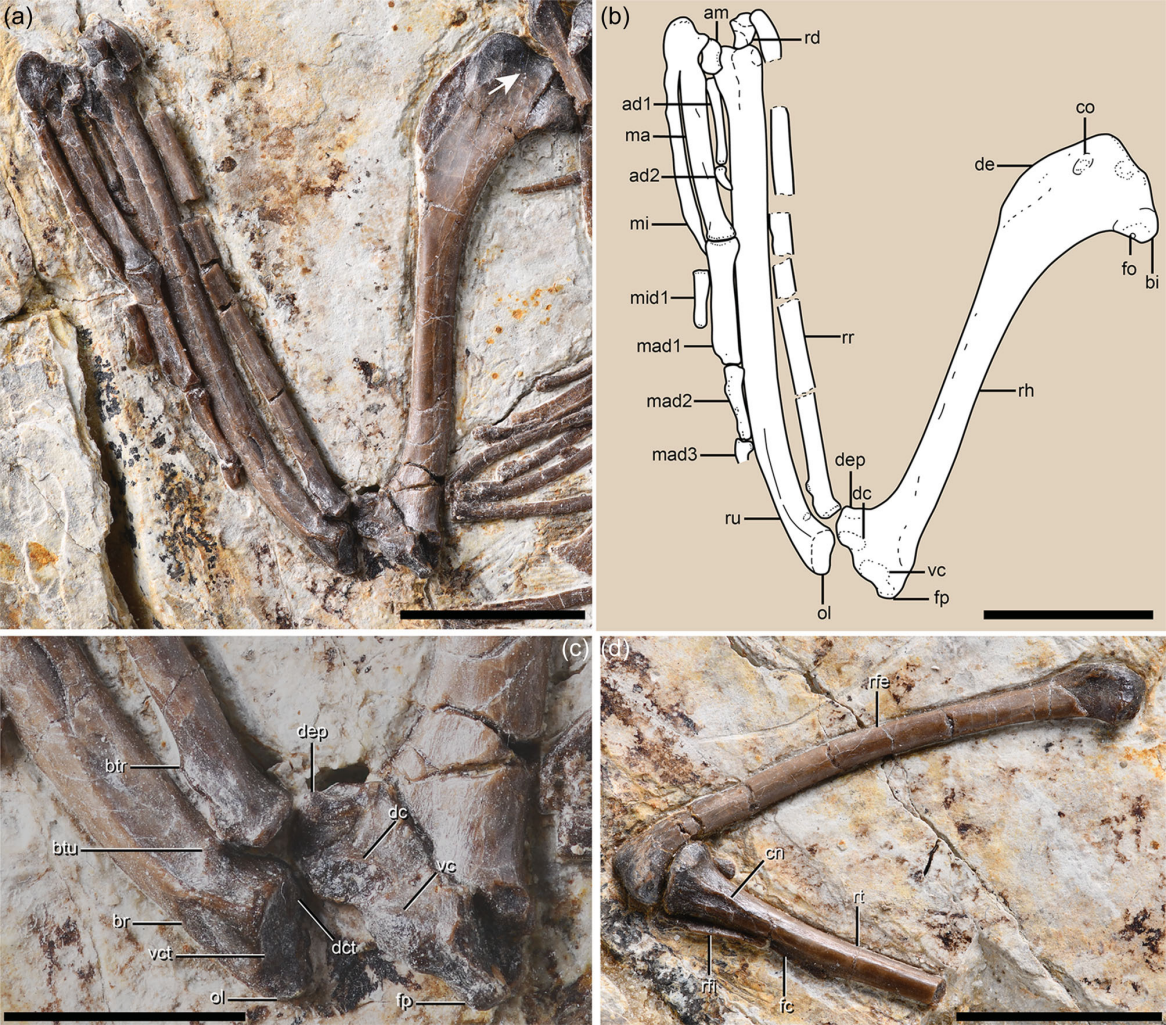
Forelimb anatomy of *Mirusavis*, IVPP V18692. (a) Photograph and (b) line drawing of the right forelimb; (c) distal end of humerus, proximal ends of ulna and radius; (d) right hindlimb. Abbreviations as in Fig. 1, except for: am, alular metacarpal; ad1 and ad2, proximal and ungual phalanges of alular digit; bi, bicipital crest; br, brachial impression; btr, bicipital tubercle of radius; btu, bicipital crest of ulna; cn, cranial cnemial crest; co, attachment for M. coracobrachialis anterior; dc, dorsal condyle; dct, dorsal cotyla; de, deltopectoral crest; dep, dorsal epicondyle; fo, fossa; fp, flexor process; ma, major metacarpal; mad1, mad2 and mad3, proximal, intermediate and ungual phalanges of major digit; mi, minor metacarpal; mid1, proximal phalanx of minor digit; ol, olecranon; vc, ventral condyle; vct, ventral cotyla. The arrow indicates the circular fossa on the proximocranial surface of the humerus. Scale bars equal 10 mm in (a), (b) and (d) and 5 mm in (c).

The humerus is twisted such that the proximal and distal ends are expanded in different planes (Fig. 2a and b), as in other enantiornithines [22,23]. The bicipital crest strongly projects cranioventrally, forming a bulbous cranial surface with respect to the proximal humerus. A pit-shaped fossa is positioned on the ventrodistal surface of the bicipital crest, as in the stem ornithuromorph *Ichthyornis* [24], whereas this fossa is more cranially located in most enantiornithines [23]. The deltopectoral crest is short, less than a quarter of the humeral length, compared to one-third in similarly sized enantiornithines such as *Cathayornis*, *Pterygornis* and *Shangyang*. The distal end of the humerus is expanded transversely and is angled ventrodistally to a greater degree than observed in other Early Cretaceous enantiornithines. The angle defined by the distal and ventral margins of the humeral shaft is 55°, which is much smaller than that of *Shangyang* (73°), *Pterygornis* (65°) and *Cathayornis* (68°). The robust ulna approaches the mediolateral width of the humerus and exceeds it in length. The olecranon is distinct, whereas it is poorly developed in most enantiornithines [19,20,22]. The ventral cotyla is flat and continuous with the dorsal cotyla, rather than being separated by a groove as in some enantiornithines (e.g. *Concornis* and *Enantiornis*) [22] or a ridge as in most crown birds [25]. A bicipital tubercle is developed on the interosseous surface of the proximal ulna (Fig. 2c), lying proximoventral to the shallow brachial impression—a structure absent in most Early Cretaceous enantiornithines except *Zhouornis* [26,27]. Proximally, the radius has a bicipital tubercle that is more pronounced than that of the ulna. The radius lacks a longitudinal groove on its interosseous surface—a feature once considered a synapomorphy of the Enantiornithes when data were limited to Late Cretaceous materials [20,22]. The major and minor metacarpals are fused proximally to the semilunate carpal, forming a carpometacarpus (Fig. 2a). The minor metacarpal is craniocaudally compressed and projects farther distally than the major metacarpal, curving onto the ventral side of the latter element. The major digit ungual is larger than that of the of alula digit, contrasting with the condition in most other enantiornithines [20,26].

The femur is slightly bowed craniocaudally and lacks a patellar groove distally, with the femoral head lost to abrasion (Fig. 2d). The medial surface of the medial condyle bears a tear-drop-shaped fossa located adjacent to the cranial margin. The tibiotarsus only has a small cranial cnemial crest that extends from the proximal margin and disappears distally proximal to the fibular crest. A lateral cnemial crest is absent. The medial margin of the proximal articular facet is elevated relative to the lateral margin, as in some taxa such as *Soroavisaurus* [22]. The fibular crest is narrow and extends along the middle third of the preserved length of the tibiotarsus. Only a proximal portion of the right fibula is preserved, revealing that the medial surface is flat rather than concave as in bohaiornithids [28] (see Supplementary Materials for additional description).

### Osteological description

**Figure 3.**
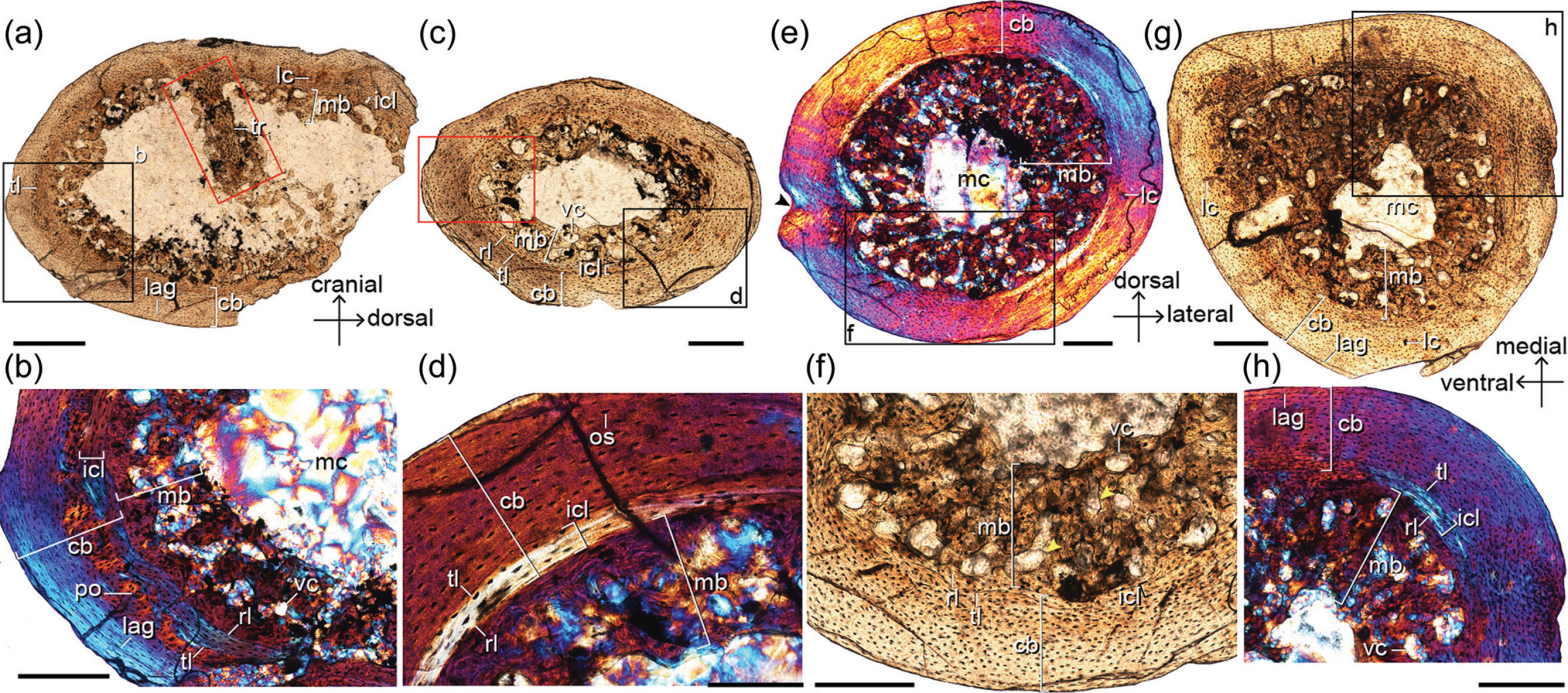
Limb-bone histology of *Mirusavis*, IVPP V18692. Thin cross sections of humerus ((a) and (b)), radius ((c) and (d)), femur ((e) and (f)) and tibiotarsus ((g) and (h)). cb, cortex bone; ilc, internal circumferential layer; lag, line of arrested growth; lc, longitudinal canal; mb, medullary bone; mc, medullary cavity; os, osteocyte; po, primary osteon; rl, resorption line; tl, tide line; tr, trabecula; vc, vascular canal. Images were taken under normal (a, c, f, g) and polarized lights (b, d, e, h), respectively. Areas in red boxes are in high magnification in Supplementary Fig. 2. Scale bars equal 400 μm in (a), 200 μm in (b), (c) and (e)–(h), and 100 μm in (d).

Petrographic cross sections have been prepared using bone samples taken from the right scapula and the right limb long bones (humerus, radius, femur and tibiotarsus) as close to the mid-diaphysis as possible (see the ‘Materials and methods’ section). The transverse cross section of the humerus reveals a large volume of endosteal woven tissue extending into the medullary cavity that can be traced circumferentially (Fig. 3a). This endosteal tissue is identified as MB based on its location and microstructure. The specimen shows no features incongruous with this identification based on the criteria established for identification of MB in fossils [7,8,29]; IVPP V18692 is clearly not growing rapidly and the periosteum is normal throughout, with no indication of pathology resembling osteopetrosis [12]. The cortical bone (CB) is composed almost entirely of parallel-fibered bone tissue and is poorly vascularized predominately by longitudinal primary osteons. The medullary surface of the CB is lined by a thin layer of avascular lamellar bone—the inner circumferential layer (ICL), which in living birds often indicates the approach of skeletal maturity (Fig. 3b) [17]. The osteocyte lacunae in the ICL are uniformly flattened and highly organized in parallel. Peripheral to the ICL and separated by a resorption line is a layer of woven bone with more globular osteocyte lacunae and a few primary osteons. No secondary

osteons are visible. One line of arrested growth (LAG) interrupts the outer cortex close to the periosteal surface on the caudal side but, due to ontogenetic osseous drift, the LAG cannot be traced circumferentially around the entire cortex. The MB is almost half the thickness of the CB and consists of well-vascularized woven tissue with large canals (Fig. 3b). The MB and CB are separated by a scalloped resorption line. Most of the canals in the humeral MB are longitudinal, in contrast to the radial morphology observed in the MB in the hindlimb of the pengornithid (IVPP V15576) [7] and possible MB in some non-avian theropods [8]. The osteocyte lacunae in the MB are generally larger and more globular than in the CB, consistently with more rapid deposition. A thick trabecula like that observed in the femur of IVPP V15576 [7] extends caudodorsally from the cranial side, nearly dividing the medullary cavity into two equal portions. This trabecula is composed of primarily parallel-fibered bone with flattened osteocyte lacunae and is itself coated in a layer of MB as in IVPP V15576.

The transverse diaphyseal section of the radius is complete and oval in shape, exhibiting minimal taphonomic distortion (Fig. 3c and d). The MB is present and nearly as thick as the CB. As in the humerus, the MB is well vascularized primarily by longitudinal canals that vary greatly in diameter and some of them appear to anastomose with one another (Supplementary Fig. 2b). An ICL is present, measuring approximately one-sixth of the CB thickness. The CB of the radius is composed of a mixture of avascular parallel-fibered and lamellar bone. Neither primary nor secondary osteons are visible in the sectioned plane. The osteocyte lacunae become progressively more flattened and their density decreases approaching the periosteum—features that suggest a decrease in the rate of bone deposition prior to death.

The mid-diaphyseal cross section of the femur is complete and contains a large amount of MB, nearly filling the entire medullary cavity (Fig. 2e and f,

and Supplementary Fig. 3a and b). The MB consists of woven tissue with large longitudinal and radial canals, such that it is more vascularized than the MB found in the humerus and radius, but similar to the MB in the femur of pengornithid IVPP V15576. The medioventral side of the cortex bears a small concavity ventrally bounded by a small protuberance. The orientations of the collagen fibers are aligned with the concavity towards the medullary cavity, suggesting this feature is not a preservational artifact. The origin of this concavity is unlikely to be pathologically derived given the absence of unusual endosteal or periosteal bone deposition that normally is associated with pathology [7,12]. Alternatively, this concavity could be the nutrient foramen.

The diaphyseal cross section of the tibiotarsus is asymmetrically oval, such that the ventromedial margin is the most acute and the medial margin appears flatter and wider than the lateral margin (Fig. 3g and h, and Supplementary Fig. 3c and d). The MB spans the circumference of the medullary cavity, exceeding the CB in thickness. The proportions are most pronounced in the mediocaudal corner of the cavity where the MB measures twice the thickness of the CB. The MB consists of well-vascularized woven tissue wherein most canals are longitudinally oriented with occasional laminar and radial canals. As in the humerus, some longitudinal canals have fused with one another. The ICL of the tibiotarsus is proportionately thinner than in other sectioned limb bones. A LAG, located just adjacent to the periosteum, can be traced circumferentially except on the cranial side.

The transverse section of the scapular blade forms a narrow rectangle housing five internal cavities. The two larger dorsal cavities contain small amounts of MB organized into thin small spicules (Supplementary Fig. 4a and b). The MB is slightly darker than the CB and is demarcated from the CB by resorption lines. The CB consists of parallel-fibered bone without any growth marks or an ICL.

**Figure 4.**
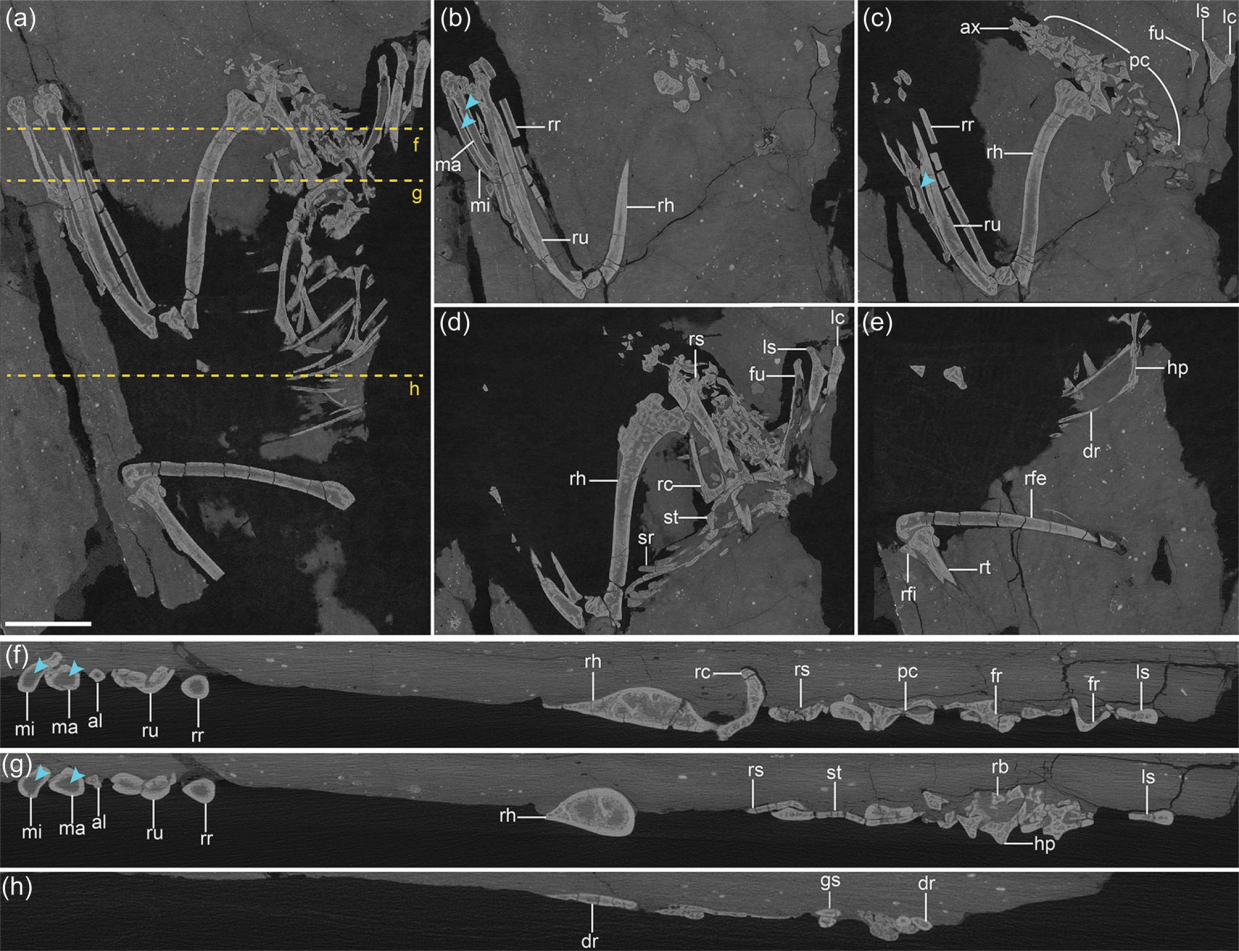
X-ray computed tomography imaging of *Mirusavis*, IVPP V18692. (a)–(e) Dorsal/ventral view of the full skeleton (a) and in different horizons ((b)–(e)), showing the distribution of medullary bone; (f)–(h) virtual cross sections (denoted by orange dashed lines in (a)) of individual elements showing the medullary bone in all elements but the metacarpals and digits (blue arrowheads). al, alular digit; ax, axis; dr, dorsal rib; fr, furcular ramus; fu, furcula; gs, gastralia; hp, hypocleidium of furcula; lc, left coracoid; ls, left scapula; ma, major metacarpal; mi, minor metacarpal; pc, post-axis cervical vertebrae; rc, right coracoid; rfe, right femur; rfi, right fibula; rh, right humerus; rr, right radius; rs, right scapula; rt, right tibiotarsus; ru, right ulna; sr, sternal rib; st, sternum. Scale bar equals 10 mm in (a); (b)–(h) are not scaled.

High-resolution computed tomography (CT) scans reveal large amounts of cancellous tissue within the cavities of most postcranial elements (Fig. 4). Specifically, in the virtual cross sections (VCSs) of the histologically sampled bones (humerus, radius, femur, tibiotarsus and scapula),

voluminous endosteal tissue, appearing slightly paler than the CB, is clearly evident lining the medullary cavities, corresponding well with the MB identified in the ground sections (Fig. 4b–h). The same endosteal tissue is also readily visible in the VCSs of the fibula, ulna, ribs, gastralia, cervical vertebrae, coracoids and sternum, which were not histologically sectioned. In light of the comparison between the ground sections produced from five elements with their VCSs, which have been used for identifying MB in previous studies [7,29,30], the paler endosteal tissues in these un-sectioned elements are interpreted as MB. No MB was detected in the metacarpals or the manual digits (Fig. 4f and g). The feet in the pengornithid IVPP V15576 and *Avimaia* IVPP V25371 were CT scanned in order to determine whether IVPP V15576 has MB in the tarsometatarsus and whether IVPP V25371 has MB in the lower leg and pedal digits, but the scans do not provide enough resolution or are too obscured by diagenesis to confirm the presence of MB in these elements (Supplementary Fig. 5).

## DISCUSSION

**Figure 5.**
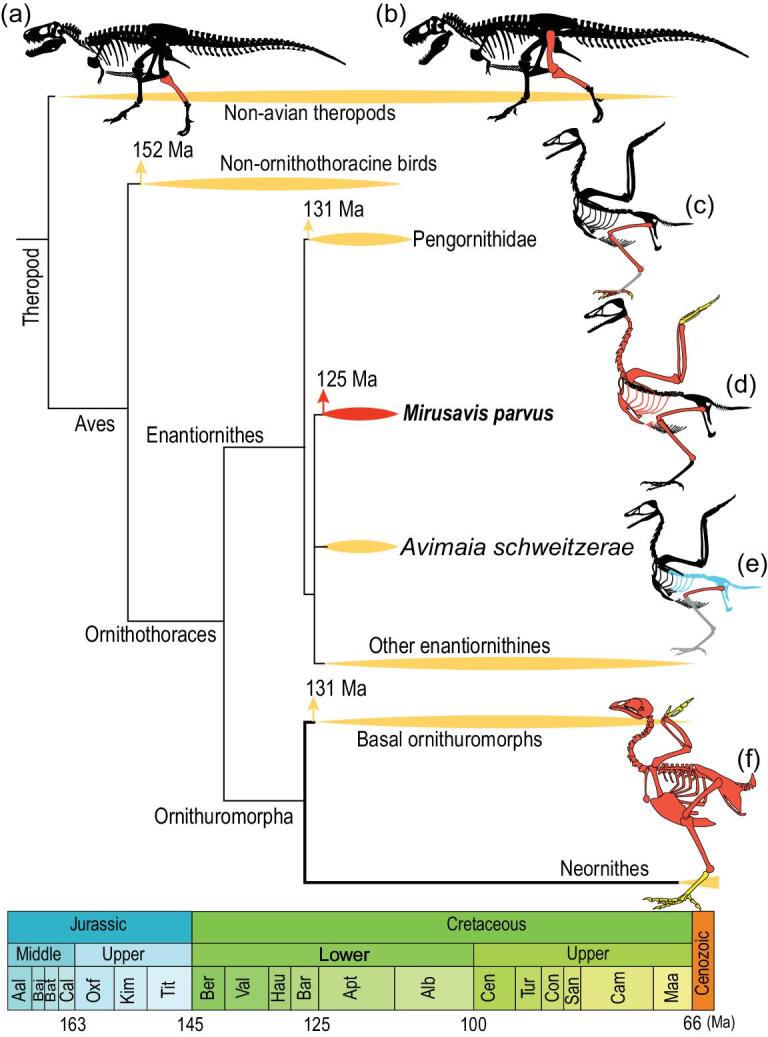
Cladogram showing the known medullary bone in theropod dinosaurs. The tree is simplified from the strict consensus recovered in present phylogenetic analysis (see Supplementary Fig. 6 for complete result) with reference to recent studies [31]. (a)–(f) Skeletal distribution of MB in *Allosaurus fragilis* ((a) Theropoda: Allosauridae) [8], *Tyrannosaurus rex* ((b) Theropoda: Tyrannosauridae) [8], Pengornithidae indet. ((c) Aves: Enantiornithes) [7], *Mirusavis parvus* ((d) Aves: Enantiornithes), *Avimaia schweitzerae* ((e) Aves: Enantiornithes) [31] and an example of modern bird *Sterna dougallii* (f) [29]. The sketches of *Allosaurus fragilis* and *Sterna dougallii* are modified from the literature [18,29]. Bones in black indicate missing, red for the presence of MB, yellow for the absence of MB, gray for ambiguity due to preservation and blue for not investigated but preserved.

Our phylogenetic analysis recovered *Mirusavis* as an enantiornithine (Fig. 5 and Supplementary Fig. 6). MB indicates that the holotype of *Mirusavis parvus* was a female and reproductively active at the time of death. However, the absence of an outer circumferential layer, which marks the termination of growth in extant birds [17], suggests it was not fully skeletally mature, corroborating the hypothesis that enantiornithines reached sexual maturity before skeletal maturity [7,31]—a growth strategy opposite to that of most crown birds [1]. IVPP V18692 provides a rare window into the physiological changes experienced by stem avians during the egg-laying cycle. Although the skeleton is not preserved in its entirety, IVPP V18692 is the most complete specimen preserving MB uncovered hitherto and thus provides important data concerning the distribution of this rare reproductive tissue early in avian evolution. MB serves as a readily available source of calcium and phosphorus for the eggshell production [3,5]. This protects the integrity of the CB, which is relatively thinner in birds compared to other egg-laying reptiles and makes bird bones potentially susceptible to failure if the full, intensive demand of calcium imposed by eggshell production was inflicted directly on the skeleton without the presence of MB [1,32]. In crown birds, MB distribution varies little intraspecifically, but shows great interspecific variation; this appears to be phylogenetically independent and largely driven by the distribution of pneumaticity and red bone marrow [29,30,33]. Highly pneumatic bones like the humerus are more likely to be devoid of MB, whereas well-vascularized elements with hematopoietic marrow normally possess large amounts of MB. As such, the skeletal distribution of MB in stem birds can also be used to infer the skeletal distribution of pneumaticity and marrow.

Although MB has been documented in several non-avian dinosaurs and Early Cretaceous birds [7–9,31,34], because nearly all previous specimens consist of partial hindlimbs or less, little is known about the actual skeletal distribution of MB in a given taxon (Fig. 5). MB is well documented in the femora and tibiae of *Tyrannosaurus rex* and several stem birds [6–9]. This is not unexpected—MB universally forms in the femur and tibia in crown birds [29,30]. The only possible exception is a complete specimen of *Confuciusornis* that reportedly has little to no MB in the tibiotarsus but preserves MB in the humerus and ulna [34]. However, the identification of MB in this specimen is considered problematic by some [7]. Collectively, the available evidence indicates the condition in which the hindlimb serves as the major reservoir of MB has been conserved along the line from non-avian dinosaurs to crown birds, further supporting reinterpretations regarding the purported MB in *Confuciusornis* [7].

Surprisingly, MB was found to be present in 92% of the preserved postcranial elements in *Mirusavis*, including delicate elements such as the ribs. A similar pattern of distribution is observed in some small passerines. Notably, MB is present in the humerus and all the cervical vertebrae—elements in which MB is less likely to form in modern birds due to the penetration of these bones by air sacs [29]. The presence of MB in the humerus and cervicals in turn suggests those elements were apneumatic in *Mirusavis*, consistently with the fact that a pneumotricipital foramen perforating the proximal humerus is limited to Late Cretaceous enantiornithines [20].

Among those modern taxa in which the distribution of MB is known, the recovered distribution of MB in *Mirusavis* is most similar to that of *Scolopax minor* (Scolopacidae), *Sterna dougallii* (Charadriiformes) and *Turdus migratorius* (Passeriformes) [29]. In modern birds the eggs of precocial taxa are generally larger, more asymmetrical and more elliptical than those of equally sized altricial species (Supplementary Fig. 7a), which places greater calcium demands on the females of precocial species [1,2]. In turn, precocial birds generally possess more extensive distribution of MB compared to altricial birds (Supplementary Fig. 7b), although the disparity is not statistically significant (*p* > 0.05; see the ‘Materials and methods’ section).

Precociality is also inferred for the Dinosauria including theropods closely related to birds [35]. All evidence thus far indicates the Enantiornithes were highly precocial, often described as superprecocial [35,36]. *Mirusavis* is estimated to have produced an egg approximately 20% of its body mass, therefore demanding large amounts of calcium during the short period in which the eggshell is formed consistent with the extensive distribution of MB documented in IVPP V18692.

MB is not documented in the pedal phalanges of neornithines [29] but is recorded in a pengornithid enantiornithine [7]. Pneumaticity aside, this suggests that the distribution of MB in stem taxa was more extensive than that of extant taxa. Unfortunately, the pedal digits are not preserved in *Mirusavis* IVPP V18692 and MB cannot be confirmed in the toes of *Avimaia* (see the ‘Materials and methods’ section). We suggest the widespread distribution of MB observed in *Mirusavis* and other enantiornithines likely represents the plesiomorphic condition or close to it. MB formation is a systemic process, so it intuitively makes sense that it would first evolve as a completely skeletal-wide feature, not limited by pneumaticity, which was low in non-neornithine dinosaurs.

MB in the hindlimb is conservative, serving as the major repository for MB in both stem and extant birds, whereas selection for increased aerial capabilities produced extensive modifications in the forelimb related to the spread of pneumaticity, in turn limiting the distribution of MB. Changes in MB distribution reflect the modularity that characterizes early avian evolution. We hypothesize that the diverse distribution patterns of MB observed among extant neornithines evolved from a complex interplay between body size, developmental strategy, ecology and pneumaticity, selecting on a systemic process whose origins are nested deep in avian evolution in order to efficiently meet the demands of each particular species.

## MATERIALS AND METHODS

### Differential diagnosis

Morphologically, *Mirusavis* most strongly resembles the similarly sized enantiornithines *Shangyang*, *Pterygornis* and *Cathayornis*, particularly with regard to sternal morphology (Supplementary Fig. 1c–f). These taxa share the presence of craniolateral processes (absent in *Cathayornis*) and lateral trabeculae with large, triangular distal expansions. However, *Mirusavis* can be distinguished from these taxa on the basis of the following features: the intermediate trabeculae are straight and lack medial deflection, whereas this process is triangular in other enantiornithines [37,38]; a well-developed bicipital tubercle is absent on the ulna in *Shangyang* [21] and *Pterygornis* [37]; the ungual of the major digit is larger than that of the alular digit, opposite to the condition in *Cathayornis* and *Pterygornis*; and the fossa on the humeral bicipital crest is more ventrally positioned relative to that of *Pterygornis* [37].

### Phylogenetic analysis

To explore the phylogenetic position of IVPP V18692, we added the new specimen and the stem pygostylian *Jinguofortis perplexus* into a comprehensive data set recently available for Mesozoic avian phylogeny [21]. The modified matrix consists of 280 morphological characters and 71 taxa, including major representatives (68 taxa) of Mesozoic birds (see Supplementary Data). Following the original study [21], 35 characters were ordered. The data set was analysed under equally weighted parsimony using the TNT v. 1.5 software package [39], with the following protocol: an unconstrained heuristic search was performed using 1000 replicates of random addition sequences followed by tree-bisection-reconnection (TBR) branch swapping, with 10 trees saved from each step; a second round of TBR search was conducted using the most parsimonious trees recovered in the previous search, collapsing zero-length branches to create polytomies. Bremer and bootstrap values were calculated to measure the support. Bremer values were obtained using the ‘Bremer script’ embedded in TNT. The absolute bootstrap values were calculated via 1000 replicates generated with the same settings as used in the primary search. The phylogenetic analysis recovered 672 most parsimonious trees with length of 1274. The strict consensus is poorly resolved, wherein *Mirusavis* forms a sister group with *Shangyang* that falls in a large polytomy within the clade Enantiornithes (Supplementary Fig. 7).

### Bone histological analysis

To investigate the histology of IVPP V18692, ground sections of bones were prepared following the standard methodologies [40]. Bone samples were taken from the right limb long bone elements (humerus, radius, femur and tibiotarsus) as close to the mid-diaphysis as possible and also from the left scapula. Bone samples were embedded in one-component resin (EXAKT Technovit 7200) and then hardened in a light polymerization device (EXAKT 520) for approximately 24 hours. Histological sections were cut transversely using an accurate circular saw (EXAKT 300CP) and were ground down using the EXAKT 400CS grinding system until the desired optical contrast was retained (the thickness of the slices in this study is approximately 40–65 μm). Histological microstructures were observed by light microscopy (Zeiss AX10) under normal and polarized light, and images were taken using a digital camera (Zeiss AxioCam MRc5).

### CT imaging

To visualize and estimate the skeletal distribution of MB, IVPP V18692 was scanned using an industrial CT scanner Phoenix v|tome|x at Yinghua Inspection&Testing, Shanghai, China. The specimen was scanned with a beam energy of 190 kV and a flux of 75 μA at a resolution of 14.8 μm per pixel. A total of 1107 slices were acquired.

In order to explore whether MB is formed in the tarsometatarsus of the pengornithid IVPP V15576 that was not examined in a previous study, the left tarsometatarsus of IVPP V15576 was scanned using an industrial CT scanner Phoenix v|tome|x at the University of New England at a resolution of 28.77 μm per pixel. The VCSs of the metatarsals II–IV exhibit relatively thick CB and possibly lack any sign of MB throughout the preserved length (Supplementary Fig. 5a–d), but the resolution is not high enough. We also scanned the right-leg and pedal digits of *Avimaia* (IVPP V25371) using the 225-kV high-resolution CT apparatus at the Key Laboratory of Vertebrate Evolution and Human Origins of Chinese Academy of Sciences, with a beam energy of 160 kV and a flux of 120 μA at a resolution of 31.37 μm per pixel. All the VCSs of the right tibiotarsus, tarsometatarsus and non-ungual pedal phalanges show no clear internal cavities (Supplementary Fig. 5f and g). The cross sections are homogenous in gray value, which is most likely resultant from the penetration of minerals during diagenesis. In addition, in *Avimaia* (IVPP V25371), the histological section of the femur shows that the MB is extremely thin [31], indicating that MB was almost entirely resorbed prior to death, which could make it harder to detect MB in the tarsometatarsus and pedal digits if it was formed.

### Skeletal distribution of MB and egg masses in modern birds

To compare the skeletal distribution of MB among modern birds across different developmental strategies (precocial, semiprecocial, semialtricial and altricial), a data set was assembled focusing on the modern species examined for MB in the Canoville *et al.* study [29]. The data set consists of 38 modern specimens representing 37 genera with female body mass ranging from 6.3 g (*Phaethornis superciliosus*) to 22 500 g (*Rhea americana intermedi*a). Information regarding the developmental strategy, egg mass and female body mass were taken from the literature (Supplementary Table 2). Where egg-mass data were unavailable, we used the egg/female body mass of their closely related and equally sized taxa. The body mass of IVPP V18692 was estimated using an equation on the basis of the femoral circumference [41]: log_10_(body mass) = 2.749 × log_10_(femoral circumference × 2^0.5^) – 1.104. We estimated the egg mass of IVPP V18692 based on an empirical equation described for precocial birds [42]: egg mass = 0.476 × (body mass^0.695^). The body and egg masses were log_10_-transformed before subsequent analyses. A linear regression between the egg mass and female body mass was conducted for altricial and precocial birds, respectively. The result shows that precocial birds have relatively heavier eggs than altricial ones (Supplementary Fig. 7a), as suggested in a previous study [43]. Although the MB is generally more extensively distributed in precocial birds (Supplementary Fig. 7b), the degree of MB distribution is not significantly different between precocial and altricial birds from a two-sample *t*-test (degrees of freedom = 33.991, *p* = 0.1516). The insignificant difference of the MB distribution between precocial and altricial birds may prove to be true, but more taxonomic sampling is needed to clarify this issue.

## Supplementary Material

nwz214_Supplemental_Files
